# Assessment of potential drug-related problems (PDRP) and clinical outcomes in bacterial meningitis patients admitted to tertiary care hospitals

**DOI:** 10.1371/journal.pone.0285171

**Published:** 2023-10-09

**Authors:** Muhammad Ali, Muhammad Harris Shoaib, Shagufta Nesar, Hira Akhtar, Saira Shahnaz, Quratulain Khan, Javaria Imran

**Affiliations:** 1 Faculty of Pharmacy and Pharmaceutical Sciences, Department of Pharmaceutics, University of Karachi, Karachi, Pakistan; 2 Niazi College of Pharmacy, Niazi Medical and Dental College, Sargodha, Pakistan; 3 Jinnah College of Pharmacy Sohail University, Karachi, Pakistan; 4 Department of Pharmaceutics, Nazeer Hussain University Karachi, Karachi, Pakistan; 5 Department of Pharmacy Practice, Nazeer Hussain University Karachi, Karachi, Pakistan; 6 Sindh Government Dispensary, Gharibabad District Central, Karachi, Pakistan; 7 NMC Specialty Hospital, Al Nahda, Dubai, UAE; Nitte University, INDIA

## Abstract

Meningitis is an important cause of morbidity and mortality in children and adults. Its treatment strategy varies with age and gender. To assess potential drug-related problems (PDRP) and clinical outcomes in bacterial meningitis patients, a multicenter, clinical, descriptive, cross-sectional prospective observational study in 120 patients admitted to different tertiary care hospitals in Karachi was conducted. It includes both males 48% and females 52% belonging from all age groups i.e. peadiatrics (01 to 12 years), adults (18 to 65 years), and geriatrics (66 to 75 years). Out of these 72 patients were admitted in the public sector and 48 patients were admitted in private sector hospitals. Nosocomial infections were developed in 41% of patients during their stay at the hospital. Potentially nephrotoxic drugs were administered to all BM patients, these drugs should be administered carefully. Majorly Ceftriaxone was administered to 86% of patients, Vancomycin 71%, and meropenem 73% whereas 68% of patients were administered piperacillin-tazobactam. Organisms involved as causative agents in the majority of patients are *Neisseria meningitides*, *Pseudomonas aeruginosa and*, *Streptococcus pneumoniae*. DRPs impacted patient clinical outcomes in presence of many other factors like comorbidities, DDIs, Nis, administration of potentially nephrotoxic drugs, and administration of watch group and reserve group antibiotics without having culture sensitivity test, even after having CST no principles of de-escalation for antibiotics were done, which is a very important factor for hospitalized patients having IV antibiotics. The mortality rate among BM patients was 66%. The majority of patients (87%) stay at the hospital was 1–10 days. The present study helped in the identification of DRPs along with some other factors affecting the clinical outcomes in patients suffering from bacterial meningitis. Healthcare professionals should receive awareness and education on the importance of CST before initiating antibiotic therapy. Pharmacist-led medication review is necessary and should be followed to avoid negative outcomes and serious consequences related to DRPs.

## Introduction

Meningitis is a serious infection of the meninges, and the membranes surrounding the brain and spinal cord. It is a dangerous disease and remains a major public health challenge. This can be caused by many different pathogens including bacteria, fungi, or viruses, but the highest global burden is seen with bacterial meningitis [1]. Bacterial and viral meningitis can be deadly in all age groups [2]. Bacterial meningitis (BM) requires immediate hospitalization [3]. Early diagnosis and treatment will prevent brain damage and death. Treatment is determined by the cause of meningitis. According to WHO, seven different types of bacteria cause bacterial meningitis [4]. Many pathogens are involved in causing bacterial meningitis but mostly it is caused by *Streptococcus pneumoniae* [5]. BM is treated with intravenous antibiotics [6]. Ceftriaxone, rifampicin and ciprofloxacin are the most effective antibiotics against *Neisseria meningitides* [7]. Initial Management Steps are taken when a patient presents with suspected acute bacterial meningitis, the physician should begin antimicrobial therapy as soon as possible whereas administration of specific antibiotic for BM depends on the bacteria involved [8]. BM is a neurologic emergency; progression to more severe disease reduces the patient’s likelihood of a full recovery, complications that are typically associated with meningitis are Seizures, hearing loss, vision loss, memory problems, migraine headaches, and brain damage [9]. Due to neurological diseases (NDs) healthcare system of developing countries faces high rate of mortality as compared to developed countries [10,11]. Care and treatment of NDs in developing countries varies depending upon the resources/facilities and needs of the country and the availability of health care personnel. Developing countries like Pakistan faces a major shortage of trained nurses, paramedical staff, and rehabilitation services related to NDs care, there are only a handful of neurologists serving large populations hence this puts a huge burden of work on the neurologists and all of this has an impact on mortality and morbidity rate of NDs patients [12]. There are many types of NDs which include stroke, meningitis, epilepsy, Alzheimer’s, cerebral palsy, migraine, and many more, many of these diseases are treated without any proper effective therapies [13]. Blood culture and lumbar puncture should be performed immediately to confirm the diagnosis. Computed tomographic (CT) scan, Cerebral spinal fluid (CSF) and Magnetic resonance imaging (MRI) can be advised by a neurologist for further confirmation of the disease [14]. The prevalence and burden of BM in Pakistan is moderate to high depending upon the rural, and urban areas and living standards of people [15]. [16] and in South Asia, the burden and incidence of BM are also high [17]. NDs are an important cause of mortality worldwide. Globally, the burden of NDs has increased over the past three decades because of the increasing population, policy makers and healthcare providers should be aware of these trends to provide adequate services [18].

Drug-related problems (DRPs) are defined as an event involving drug therapy that potentially interferes with the desired health outcomes, common problems faced by patients due to DRPs are inappropriate prescribing, drug–drug interactions (DDIs), non-adherence, and adverse drug reactions [19]. DRPs are more prevalent in hospital settings and they impacted the patient quality of life, it is now necessary that drugs to be used rationally [20]. Pharmacotherapy monitoring should be done under the supervision of a pharmacist to avoid negative outcomes caused by DRPs [21]. DRPs should be resolved appropriately to avoid serious consequences and pharmacist-led interventions will make sure safe and effective pharmacotherapy [22]. In BM patients mostly antibiotics are administered in combination with various other drugs, so monitoring of DRPs should be done properly by clinical pharmacists [23].

This current study primarily aimed at the assessment of drugs administered to hospitalized BM patients in terms of safety and efficacy. Potential drug related problems (PDRP) along with the factors affecting the mortality rate and clinical outcomes among BM patients were also assessed using some points from Pharmaceutical Care Network Europe (PCNE) Classification Version 9.1, a tool or classification scheme used for assessment of DRPs [24].

## Methods

### Study design and sample

This study was a multicenter, clinical, cross-sectional prospective observational study including 120 BM patients admitted in one public and two private tertiary care hospitals. The study sample included BM patients admitted either in the Neurology ward, medical ward, medical or surgical ICU, and isolation ward of public and private tertiary care hospitals in Karachi, Pakistan. The study sample included all the patient’s fulfilling inclusion and exclusion criteria. The study was conducted from September 2021 to August 2022.

### Ethical approval

The current study protocol was approved by the Advanced studies and research board (ASRB) of the University of Karachi, Karachi, Pakistan (Letter, ASRB/No./06314/Pharm.) and the Ethical Review Committee (ERC) of Sohail University, Karachi, Pakistan (Letter, Protocol I #:000160/22). Patient data was then collected from affiliated teaching hospitals of the University of Karachi, Karachi, Pakistan, and Sohail University, Karachi, Pakistan.

### Patient data collection and clinical evaluation

Patient data were collected from patients’ daily data files after taking their consent using a developed/adapted checklist. Patient’s age, gender, clinical outcome, major disease, hospital stay, and hospital sector were recorded. Other data information included nosocomial infections, culture sensitivity tests, drug therapy (antibiotics, antivirals, antipyretics, antifungals, antiepileptics, antihypertensives, antidiabetics, PPIs, NSAIDs, etc), lab diagnostics and drug-related problems were noted using an adapted checklist. The renal function was assessed using the Cockroft-Gault equation. Drug-drug interactions were checked using Stockley’s drug interaction [25,26]. Clinical outcomes were determined as death or discharge of the patient on the last day of the patient stay at the hospital. Rational antibiotic therapy was determined using the results of a Blood culture sensitivity test (CST) and the American Society of Infectious Diseases (IDSA) guidelines. Antibiotics and other drug therapy initiated in BM patients were also studied and analyzed using the National Essential Medicine List (NEML) of Pakistan [27].

### Inclusion and exclusion criteria

Patients admitted in the neurology ward, medical ward, surgical or medical ICU, and isolation ward suffering from BM. Special Personnel protective equipment (PPE) was used for the assessment of BM patients data admitted in isolation wards [28,29] Patients from both genders were included and classified as paediatrics (01 to 12 years), adults (18 to 65 years), and geriatrics (66 to 75 years), and patients having a total length of hospital stay less than 01 months were included.

Patients less than one year of age, patients greater than 75 years of age, patients having length of hospital stay greater than 30 days, and patients having length of hospital stay less than 2 days were excluded.

### Study tool (evidence-based clinical checklist)

An evidence-based clinical checklist was developed after an extensive literature review and adapting some points from PCNE classification and it was employed as a study tool (see S1 File). This tool was used to evaluate patients and to determine potential drug-related problems (PDRP), and their causes (including possible causes for potential problems) [24,30]. Individual patient clinical evaluation was carried out using the developed checklist along with clinical outcomes in terms of efficacy and safety of the drug therapy. Data that was used included the patient’s age, gender, clinical outcome, major disease, hospital stay, and hospital sector. Other data information included nosocomial infections like sepsis, fever, CAP, VAP etc, culture sensitivity test, drug therapy (antibiotics, antivirals, antipyretics, antifungals, antiepileptics, antihypertensives, antidiabetics, PPIs, NSAIDs, etc), lab diagnostics like Complete blood count (CBC), CST, Serum creatinine, Lipid profile, and electrolytes, etc.

### Statistical analysis

All the data collected through the clinical checklist was transcribed on a spreadsheet in SPSS (Statistical Package for the Social Sciences), Version 25 after carefully defining all the variables under study. The analysis was performed in two steps including a descriptive analysis in which the patient clinical situation and various variables were studied for their respective frequencies, percentages, and measure of central tendencies. Later, in the second step, inferential statistics were employed to determine the associations among patient outcomes and therapy-related variables, clinical outcomes, and mortality rate using Pearson’s chi-squared test. P-value <0.05 was considered statistically significant.

## Results

**Fig 1:**
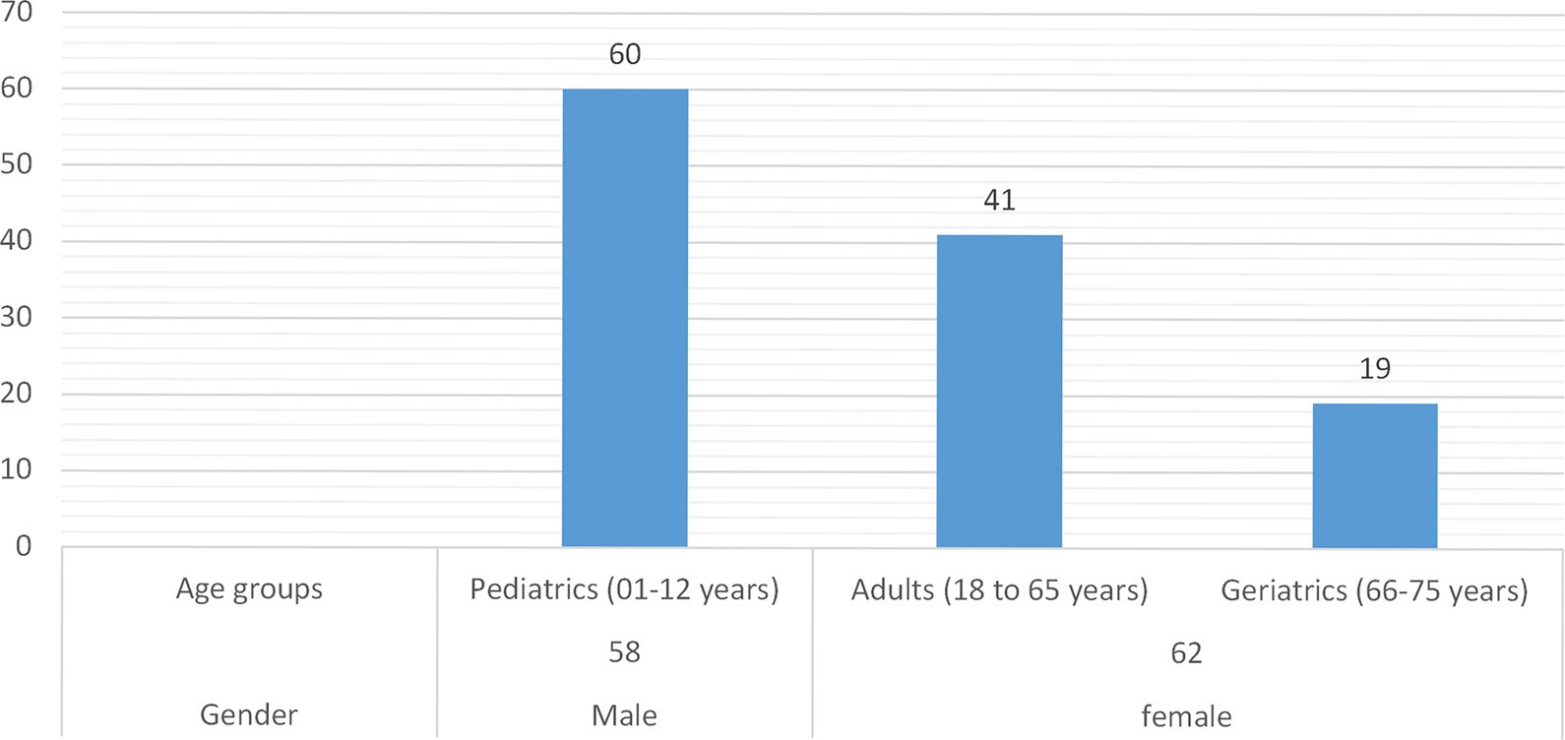
Gender and age groups of patients suffering from BM.

**Fig 2:**
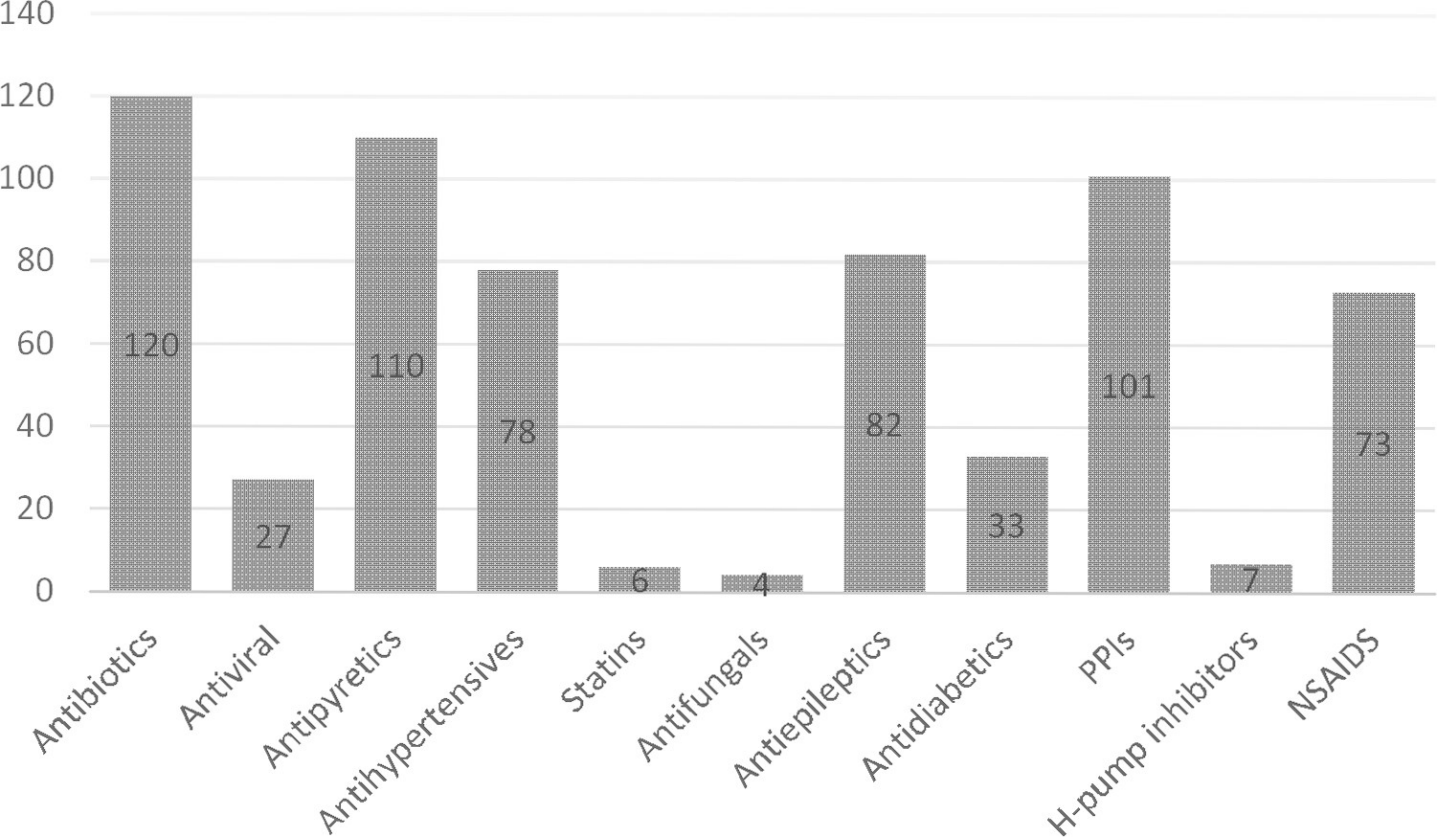
Classification of drugs prescribed/administered to hospitalized BM patients.

**Fig 3:**
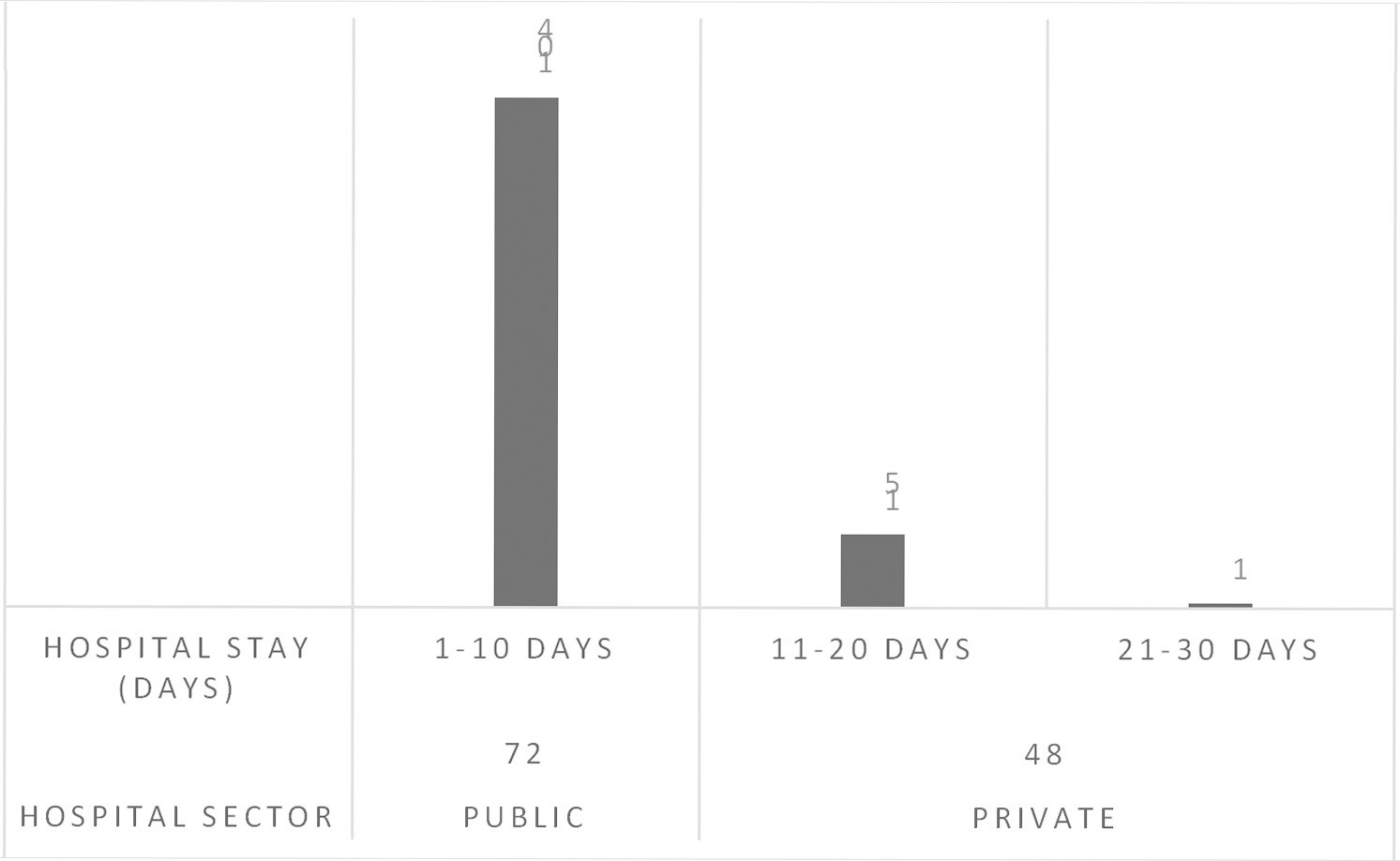
Graph showing Hospital sector and Hospital stay days of BM patients.

**Fig 4:**
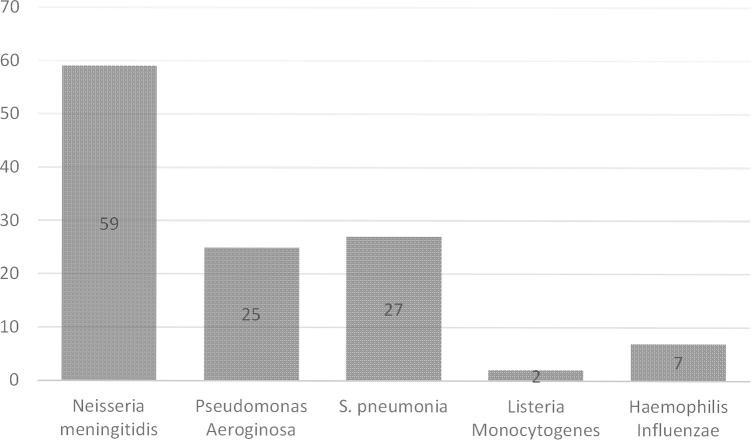
Frequency of Microorganisms causing bacterial meningitis.

**Fig 5:**
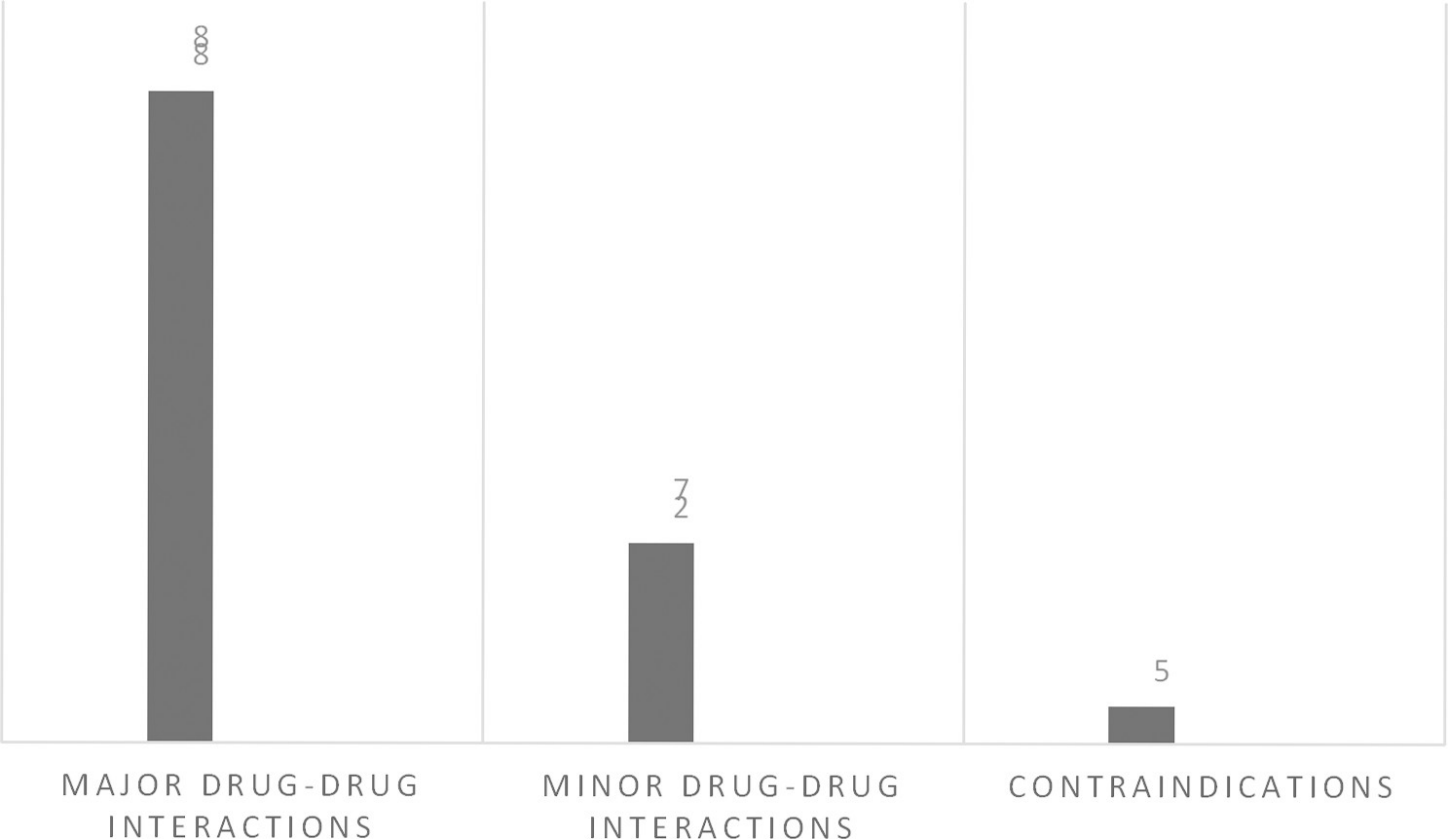
Frequency of Drug-drug interactions occurred due to multiple drug therapy in BM patients.

In this study total of 120 patients suffering from BM including both males 48% and females 52% were assessed. Sixty patients were from the peadiatrics age group (01 to 12 years), 41 patients were from the adults age group (18 to 65 years) and 19 patients were from the geriatrics age group (66 to 75 years) as presented in Fig 1. A high mortality rate of 66% was seen in this study. Multiple drug therapy was prescribed and administered to hospitalized patients, the classification of drugs is mentioned in Fig 2. The majority of patients (87%) stay at the hospital was 1–10 days. Data were collected randomly from both public (60%) and private (40%) sector hospitals as illustrated in Fig 3. Antibiotics were also classified according to the national essential medicine list (NEML) into three groups i.e. access group antibiotics, watch group antibiotics, and reserve group antibiotics. Antibiotics administered to BM patients according to NEML and their association with clinical outcomes of BM patients is provided in Table 2. Various microorganisms were seen as causative agents of BM. *Neisseria meningitides*, *Pseudomonas aeruginosa and*, *Streptococcus pneumonia* were found in majority of BM patients as demonstrated in Fig 4. In Fig 5, the frequency of DDIs is provided. All BM patients were administered potentially nephrotoxic drugs, status of potentially nephrotoxic drugs administered to compromised BM patients and their statistical associations are present in Table 1. Antibiotics administered to BM patients according to NEML and their statistical association with clinical outcomes of BM patients is described in Table 2.

**Table 1:** Status of potentially nephrotoxic drugs administered to renally compromised BM patients.

Drugs	Status of Renally compromised bacterial meningitis patients (n = 49.2% patients)		P-value
	Yes	No	
**Piperacillin-Tazobactum**	43	38	0.216
**Vancomycin**	41	44	0.750
**Ceftriaxone**	51	52	0.851
**Amikacin**	18	11	0.110
**Omeprazole**	44	51	0.223
**Levetiracetum**	46	40	0.132
**Dexamethasone**	49	58	**0.034**
**Acyclovir**	19	17	0.604
**Ciprofloxcin**	29	24	0.279
**Paracetamol**	38	42	0.606
**Diclofenac Sodium**	13	19	0.259
**Gentamicin**	06	10	0.316
**Cefixime**	09	10	0.864
**Levofloxacin**	08	10	0.664
**Moxifloxacin**	03	05	0.094
**Ibuprofen**	08	16	0.083
**Cefoperazone-Sulbactum**	09	05	0.550
**Co-Amoxiclav**	11	01	**0.000**
**Azithromycin**	17	14	0.463

**Table 2:** Antibiotics administered to BM patients according to NEML and their association with clinical outcomes of BM patients.

Antibiotics administered according to NEML	Clinical Outcomes		P-value
	Death/Expired	Discharged	
**Access group Antibiotics administration**	72	31	**0.021**
**Watch group Antibiotics administration**	79	39	**0.048**
**Reserve group Antibiotic administration**	65	28	0.082

Statistical Association between DDIs and Clinical outcomes of BM patients are shown in Table 3. DDIs are classified as major, minor and serious DDIs. The Association among DDIs, Nis, and Comorbidities among BM patients is provided in Table 4. Nosocomial infections faced by BM patients while their stay at the hospital was fever, sepsis, Ventilator-associated pneumonia (VAP), Community acquired pneumonia (CAP), Urinary tract infections, and skin infections. The majority of the BM patients were already suffering from hypertension and diabetes. The statistical association between various clinical scenarios faced by BM patients and their clinical outcomes is shown in Table 5. A culture sensitivity test was only done in 32% of BM patients. Statistical association among DRPs and clinical outcomes of BM patients is mentioned in Table 6. Table 6 is based on points taken from PCNE classification V 9.1. In Table 7, the same PCNE classification is used and the association between DRPs and Nosocomial Infections faced by BM patients is discussed. As mortality was seen in BM patients so the percentage Mortality rate of BM patients in different clinical Scenarios/Conditions is shown in Table 8.

**Table 3:** Association between Drug-drug Interactions and Clinical outcomes of BM patients.

**Drug-Drug Interactions**	**Clinical Outcomes of Patients**		**Total (n)**	**P-Value**
	**Death/Expired**	**Discharged**		**0.081**
**Major Drug-drug interaction**	62	26	88	
**Minor Drug-drug interaction**	13	14	27	
**Serious/Contraindications**	04	01	05	

p-value 0.005

**Table 4:** Association between DDI, NI and Comorbidities among BM patients.

Clinical Variable	Drug-Drug Interactions	Frequency	P-Value
**Nosocomial Infections**	Major Drug-drug interaction	32	**0.012**
	Minor Drug-drug interaction	06	
	Serious/Contraindications	05	
**Comorbidities** **(Hypertension & Diabetes)**	Major Drug-drug interaction	67	**0.000**
	Minor Drug-drug interaction	04	
	Serious/Contraindications	03	

**Table 5:** Association between various clinical scenarios faced by BM patients and their clinical outcomes.

Clinical Variable/Scenario	Clinical Outcomes of Patients		Total (n)	P-value
	Death/Expired	Discharged		
**Nosocomial Infections**	38	11	49	**0.025**
**Major Drug-drug interaction**	62	26	88	0.081
**Comorbidities**	58	16	74	**0.000**
**No Culture Sensitivity Test**	55	27	82	0.674
**SARS-COV 2 virus**	10	04	14	0.639

**Table 6:** Association between DRPs and clinical outcomes of BM patients.

Primary Domain	Cause	Frequency of DRPs	Clinical Outcomes		P-value
			Death/Expired	Discharge	
**Drug Selection**	Inappropriate drug according to guidelines/formulary	30	20	10	0.776
	Inappropriate combination of drugs, or drugs and herbal medications, or drugs and dietary supplements	39	24	15	
	Appropriate drug according to guidelines/formulary	51	35	16	
**Dose Selection**	Dosage regimen not frequent enough	37	22	07	0.463
	Dosage regimen too frequent	62	44	18	
	Dose timing instructions wrong, unclear or missing	21	13	08	
**Treatment Duration**	Duration of treatment too short	44	31	13	0.656
	Duration of treatment too long	17	10	07	
	Proper Duration of treatment	59	38	21	
**Dispensing**	Prescribed drug not available	36	31	05	**0.000**
	Necessary information not provided or incorrect advice provided	54	37	17	
**Drug use process**	Inappropriate timing of administration or dosing intervals	37	27	10	0.321
	Wrong drug administered	27	19	08	
	Appropriate timing of administration or dosing intervals	56	33	23	

**PCNE Classification V 9.1**^**24, 63**^.

**Table 7:** Association between DRPs and Nosocomial Infections faced by BM patients.

Primary Domain	Cause	Frequency of DRPs	Nosocomial Infections		P-value
			Yes	No	
**Drug Selection**	Inappropriate drug according to guidelines/formulary	30	14	16	0.085
	Inappropriate combination of drugs, or drugs and herbal medications, or drugs and dietary supplements	39	20	19	
	Appropriate drug according to guidelines/formulary	51	15	36	
**Dose Selection**	Dosage regimen not frequent enough	37	09	28	**0.031**
	Dosage regimen too frequent	62	28	34	
	Dose timing instructions wrong, unclear or missing	21	12	09	
**Treatment Duration**	Duration of treatment too short	44	14	30	**0.020**
	Duration of treatment too long	17	12	03	
	Proper Duration of treatment	59	23	36	
**Dispensing**	Prescribed drug not available	36	17	19	0.079
	Necessary information not provided or incorrect advice provided	54	25	29	
**Drug use process**	Inappropriate timing of administration or dosing intervals	37	16	21	0.119
	Wrong drug administered	27	15	12	
	Appropriate timing of administration or dosing intervals	56	18	38	

**PCNE Classification, V 9.1**^**24, 63**^.

**Table 8:** Mortality rate of BM patients in different clinical Scenarios/Conditions.

**Clinical Variable**	**Death/Mortality rate (%)**
Comorbidities	78.4
Nosocomial Infections	78.0
Major drug-drug interactions	69.0
Empiric therapy not administered	71.0
No CST performed	67.1
SARS-COV-2 (COVID-19)	71.4
Renally compromised patients	73.0
Steroids Misuse	67.3

## Discussions

In the current study, multiple drug therapy was prescribed and administered to hospitalized BM patients as mentioned in Fig 2, we have also observed this trend in previous studies as well [31]. Antibiotics were also classified according to National essential medicine list (NEML), so that the safety and efficacy of antibiotics can be checked or correlated with the clinical outcomes of patients. Antibiotics administered to BM patients according to NEML especially watch group and access group caused mortality rate due to various factors and their association with clinical outcomes of BM patients is mentioned statistically in Table 2. In this current study, 25.8% of patients were prescribed antibiotics according to guidelines [32,33]. No principles of de-escalation for antibiotics were done after the culture sensitivity test (CST), which is very important for hospitalized patients having IV antibiotics [34]. Certain other studies also focused on the rational use of antibiotics according to guidelines and also discussed the clinical outcomes of patients having IV antibiotics and now there is a need for an antibiotic stewardship programs for betterment of hospitalized patients [35].

During this study period, 41% of the patients developed Nis during their stay at the hospital. Among the patients having Nis, 27% of patients were administered antibiotics according to IDSA guidelines. A high mortality rate (78%) was found in patients having Nis. The mortality rate in this study among nosocomial infections patients was 78% not so high as compared to previous studies [35,36]. Mortality in BM patients having comorbidities (hypertension and diabetes) was also high in this current study (78.3%), hypertension is in fact a triggering factor for various complications like stroke [37]. Hypertension and diabetes are the risk factors for patients suffering from bacterial meningitis and stroke [38]. The study was carried out in medical and neurology wards also including medical and surgical ICUs, where patients are admitted due to multiple critical reasons [39]. The important factor while initiating a drug therapy is that the route of excretion or elimination and metabolism of the drug and the renal profile of the patients should be considered [40] Potentially nephrotoxic drugs were administered to BM patients who were renally compromised, these drugs needed to be avoided or administered carefully among renally compromised patients as mentioned previously in Table 1. Since a majority of the drugs are excreted through kidneys, assessing the kidney function is a key factor in ensuring the administration of safe and effective therapy [41].

In the current study, we determined that 49.2% of the patients were renally compromised, and they needed careful administration of renally excreted drugs or narrow therapeutic index drugs as due to presence of comorbidities, extreme age above 60 years and Nis chances of drug-induced nephrotoxicity is increased [42–44]. Renally compromised patients were determined using the values of serum creatinine (cockroft-Gault equation). Administration of steroids without dose adjustment is dangerous as far as renally compromised patients are concerned, yes steroids are given to BM patients but they are potentially nephrotoxic so they might cause drug-induced nephrotoxicity and may lead to mortality [45,46].

Majorly Ceftriaxone was administered to 86% of patients, vancomycin 71%, meropenem 73% whereas 68% of patients were administered piperacillin-tazobactum whereas other antibiotics administered included ciprofloxacin 44.2%, metronidazole 41%, levofloxacin and cefixime 15% & 16%, gentamicin 13.3%, cefoperazone-sulbactum 12% and azithromycin 26%. The number of various plans while prescribing and administering antibiotics were studied including CST performed in 38 patients, therapy changed after CST in 38 patients, and antibiotics administration according to guidelines initiated in 31 patients [47]. Antibiotics were evaluated according to WHO NEML as Access, Watch, and reserve group antibiotics [48,49]. Drug regulatory of Pakistan (DRAP) has also adopted that list [50]. Administration of watch group and reserve group antibiotics impacted the clinical outcomes of patients as many of these antibiotics are potentially nephrotoxic and should be used upon CST report [51]. Inappropriate combination of drugs, or drugs and herbal medications, or drugs and dietary supplements (33%) were seen, drugs included antibiotics, PPIs, antipyretics, antiepileptics, antihypertensives, statins, NSAIDs, antidiabetics, antivirals, H2 inhibitors, and antifungals, previous studies only mentioned antipyretics, steroids and antibiotics involved in DRPs [52].

Bacterial meningitis is caused by various organisms, according to our data majority of BM was caused by *Neisseria meningitides* in 59 patients, *Pseudomonas aeruginosa* in 25 patients, *S*. *pneumonia* in 27 patients, *Listeria Monocytogenes* 02 patients, *Haemophilus influenzae* in 07 patients. Previous studies also demonstrated that various causative agents were involved in causing bacterial, viral, and fungal meningitis [53,54]. A high mortality rate was seen in the case of *Neisseria meningitides 75%*, *Pseudomonas Aeroginosa 72%*, *and in the case of S*. *pneumonia 52%*. Mortality due to major drug-drug interactions was found high in our data i.e. 69% as compared to previous studies [55,56]. Many other DDIs were seen in patients due to polypharmacy [57]. In our study as we have mentioned that multiple drug therapy was administered to patients so it was observed that one of the serious interactions including fatal particulate precipitation occurred in the lungs due to the co-administration of ceftriaxone along with calcium containing intravenous solutions. Due to comorbidities like hypertension and diabetes rate of mortality increased (78.4%) much higher than previously reported studies [58,59]. SARS-COV-2 (COVID-19) was diagnosed in 14 BM patients and caused maximum mortality i.e. 71.42% [60]. Various Nis were found in BM patients e.g. sepsis was found in 20.4% of BM patients, community acquired pneumonia (CAP) was found in 51.02% of patients, Ventilator-associated pneumonia (VAP) was present in 29% of patients and fever caused mortality and one of the factors influencing changes in drug therapy, fever was noted in 89% BM patients and were administered antipyretics drugs [61]. Culture sensitivity test (CST) is necessary while administering and prescribing antibiotics. Not having CST caused high mortality rate in BM patients [62]. In Table 8, we have shown the various factors affecting mortality and morbidity in BM patients admitted to tertiary care hospitals.

Drug related problems were studied using PCNE classification V. 9.1 and their associations were analyzed statistically against clinical outcomes and Nis, association between dose selection, dispensing and treatment duration with Nis and clinical outcomes were statistically significant as mentioned in Tables 6 & 7. This current study is the first study to the best of our knowledge to use this PCNE classification for the assessment of DRPs in hospitalized BM patients. DRPs caused many problems in hospitalized BM patients, they not only caused DDIs but also effected the patient’s morbidity and mortality rate [63]. DRPs have an impact on patient quality of life and mortality rate in our study DRPs impacted patient clinical outcomes in presence of various other factors like comorbidities, DDIs, Nis, administration of potentially nephrotoxic drugs, and administration of watch group and reserve group antibiotics without having CST. Now our point of focus is that specialized clinical pharmacists and other healthcare professionals having experience in infectious disease management are needed to supervise the therapeutic interventions in such scenarios as their role is of utmost importance [64].

## Conclusion

This study was helpful in identifying DRPs along with various factors affecting the clinical outcomes in terms of morbidity and mortality in the patients suffering from BM. After the determination of DRPs and other related factors, it was observed that this study will help healthcare professionals to determine the extent to which proper clinical guidelines should be followed and required monitoring should be done. This study will also help practitioners and policymakers to make new decisions based on the results. Pharmacist-led medication review by intervening with the doctor at the patient bed site is necessary and should be followed to avoid negative outcomes and serious consequences related to DRPs along with antibiotic bombardment and steroid misuse as they have an impact on patient quality of life, prolonged hospital stay, expensive treatment and mortality rate in the developing countries. Meanwhile, awareness and education should be given to healthcare professionals regarding the importance of CST before the initiation of antibiotic therapy in hospitalized BM patients.

## Supporting information

S1 Raw data (ZIP)

S1 File (DOCX)

## Abbreviations

^1^BM: Bacterial meningitis
^2^DRPs: Drug related problems
^3^CST: Culture sensitivity test
^4^NDs: Neurological disorders
^5^DDIs: Drug-drug interactions
^6^NEML: National essential medicine list of Pakistan
^7^DRAP: Drug regulatory of Pakistan
^8^Nis: Nosocomial infections

## References

[1] Organization WH. Standardized treatment of bacterial meningitis in Africa in epidemic and non epidemic situations. World Health Organization, 2007.

[2] Jawaid A, Bano S, Haque A, Arif KJJOCOP, Pakistan S. Frequency and outcome of meningitis in pediatric intensive care unit of Pakistan. 2016;26(8):716. 27539773

[3] McMillan DA, Lin CY, Aronin SI, Quagliarello VJJCID. Community-acquired bacterial meningitis in adults: categorization of causes and timing of death. 2001;33(7):969–75. 10.1086/322612 11528567

[4] Oordt-Speets AM, Bolijn R, van Hoorn RC, Bhavsar A, Kyaw MHJPO. Global etiology of bacterial meningitis: a systematic review and meta-analysis. 2018;13(6):e0198772. doi: 10.1371/journal.pone.0198772 29889859 PMC5995389

[5] Koelman D, Brouwer M, van de Beek DJCM, Infection. Resurgence of pneumococcal meningitis in Europe and Northern America. 2020;26(2):199–204. 10.1016/j.cmi.2019.04.032 31100424

[6] Hasbun R, Aronin SI, Quagliarello VJJCt. Treatment of bacterial meningitis. 1999;25(2):73–81. 10.1007/BF02889599 10091011

[7] Prasad K, Karlupia NJRm. Prevention of bacterial meningitis: an overview of Cochrane systematic reviews. 2007;101(10):2037–43. doi: 10.1016/j.rmed.2007.06.030 17706408

[8] Davis LEJCLLIN. Acute bacterial meningitis. 2018;24(5):1264–83. doi: 10.1212/CON.0000000000000660 30273239

[9] Rizk MZ, Fouad GI, Aly HF. Neurological Disorders: Caus. 2018.

[10] Hussain G, Shahzad A, Anwar H, Mahmood Baig S, Shabbir A, De Aaguilar J-lGJPJoNS. Neurological disorder burden in faisalabad, punjab-pakistan: data from the major tertiary carecenters of the city. 2017;12(3):3–10.

[11] Kim KSJTLID. Acute bacterial meningitis in infants and children. 2010;10(1):32–42. doi: 10.1016/S1473-3099(09)70306-8 20129147

[12] Singhal B, Khadilkar SVJHocn. Neurology in the developing world. 2014;121:1773–82. doi: 10.1016/B978-0-7020-4088-7.00114-0 24365446

[13] Hussain G, Rasul A, Anwar H, Sohail MU, Kamran SKS, Baig SM, et al. Epidemiological data of neurological disorders in Pakistan and neighboring countries: a review. 2017;12(4):52–70.

[14] Salekeen S, Mahmood K, Naqvi IH, Baig MY, Akhter ST, Abbasi AJH. Clinical course, complications and predictors of mortality in patients with tuberculous meningitis—an experience of fifty two cases at Civil Hospital Karachi, Pakistan. 2013;31:59.6. 23757980

[15] Rashid M, Iqbal Bano AHJIJFS. Prevalence of Common Infectious Diseases in Paediatric Age Group Admitted in Children’s Hospital Lahore, Pakistan. 2017;1(1):19–28.

[16] Siyal H, Jamali AN, Qambrani ZAJTPMJ. Prevalence of pyogenic meningitis in a Tertiary Care Hospital of Sindh. 2020;27(10):2117–21.

[17] Ali M, Chang BA, Johnson KW, Morris SKJV. Incidence and aetiology of bacterial meningitis among children aged 1–59 months in South Asia: systematic review and meta-analysis. 2018;36(39):5846–57. 10.1016/j.vaccine.2018.07.037 30145101

[18] Karikari TK, Charway-Felli A, Höglund K, Blennow K, Zetterberg HJFIN. Commentary: global, regional, and national burden of neurological disorders during 1990–2015: a systematic analysis for the Global Burden of Disease Study 2015. 2018;9:201. doi: 10.3389/fneur.2018.00201 29651272 PMC5885159

[19] Eichenberger PM, Lampert ML, Kahmann IV, Van Mil J, Hersberger KEJPW, science. Classification of drug-related problems with new prescriptions using a modified PCNE classification system. 2010;32(3):362–72. 10.1007/s11096-010-9377-x 20229029

[20] Andreazza RS, De Castro MS, Köche PS, Heineck IJGS. Causes of drug-related problems in the emergency room of a hospital in southern Brazil. 2011;25(6):501–6. doi: 10.1016/j.gaceta.2011.05.016 21835509

[21] Comite De Consenso G-UJAP. Third Consensus of Granada on Drug Related Problems and Negative Outcomes associated with Medication. 2007;48(1):5–17.

[22] Nicolas A, Eickhoff C, Griese N, Schulz MJIjocp. Drug-related problems in prescribed medicines in Germany at the time of dispensing. 2013;35(3):476–82. doi: 10.1007/s11096-013-9769-9 23605073

[23] Ali I, Khan J, Khan AUJAOPPV. Need of advance clinical pharmacy services: A case study from Pakistan. 2015;6(3).

[24] Parsels KA, Kufel WD, Burgess J, Seabury RW, Mahapatra R, Miller CD, et al. Hospital discharge: An opportune time for antimicrobial stewardship. 2022;56(8):869–77. doi: 10.1177/10600280211052677 34738475

[25] Preston CL. Stockley’s drug interactions: pharmaceutical Press London; 2016.

[26] Baxter K, Preston CL. Stockley’s drug interactions: Pharmaceutical Press London; 2010.

[27] Organization WH. The selection and use of essential medicines: report of the WHO Expert Committee on Selection and Use of Essential Medicines, 2019 (including the 21^st^ WHO Model List of Essential Medicines and the 7^th^ WHO Model List of Essential Medicines for Children). 2019.

[28] Baloh J, Reisinger HS, Dukes K, da Silva JP, Salehi HP, Ward M, et al. Healthcare workers’ strategies for doffing personal protective equipment. 2019;69(Supplement_3):S192–S8. doi: 10.1093/cid/ciz613 31517970 PMC6743502

[29] Alomi YA, Alyousef A, Islam MM, Almadany MH, Almana FA, Badawoud EM, et al. Pharmacy Infection Control: Basic Hygiene for Pharmacy Staff. 2022;8(2).

[30] Farrukh MJ, Hisham SA, Bin Zainal ZA. Pharmaceutical care issues in patients with atrial fibrillation receiving thromboprophylaxis. Am J Pharmacol Sci. 2014;5:12–6.

[31] Hoffman O, Weber JRJTaind. Pathophysiology and treatment of bacterial meningitis. 2009;2(6):401–12. 10.1177/1756285609337975 PMC3002609 21180625

[32] Tunkel AR, Hasbun R, Bhimraj A, Byers K, Kaplan SL, Scheld WM, et al. 2017 Infectious Diseases Society of America’s clinical practice guidelines for healthcare-associated ventriculitis and meningitis. 2017;64(6):e34–e65. doi: 10.1093/cid/ciw861 28203777 PMC5848239

[33] Onakpoya IJ, Walker AS, Tan PS, Spencer EA, Gbinigie OA, Cook J, et al. Overview of systematic reviews assessing the evidence for shorter versus longer duration antibiotic treatment for bacterial infections in secondary care. 2018;13(3):e0194858. doi: 10.1371/journal.pone.0194858 29590188 PMC5874047

[34] Masterton RGJCCC. Antibiotic de-escalation. 2011;27(1):149–62. doi: 10.1016/j.ccc.2010.09.009 21144991

[35] Ali M, Naureen H, Tariq MH, Farrukh MJ, Usman A, Khattak S, et al. Rational use of antibiotics in an intensive care unit: a retrospective study of the impact on clinical outcomes and mortality rate. 2019;12:493. 10.2147/IDR.S187836 PMC6396654 30881054

[36] Pittet D, Tarara D, Wenzel RPJSOA. Nosocomial Bloodstream Infection in Critically Ill Adults: Excess Length of Stay, Extra Costs, and Attributable Mortality. 1994;38(6):361. 10.1001/jama.271.20.1598 8182812

[37] Deresse B, Shaweno DJJOTNS. Epidemiology and in-hospital outcome of stroke in South Ethiopia. 2015;355(1–2):138–42. doi: 10.1016/j.jns.2015.06.001 26059446

[38] Wasay M, Khan M, Farooq S, Khowaja ZA, Bawa ZA, Mansoor Ali S, et al. Frequency and impact of cerebral infarctions in patients with tuberculous meningitis. 2018;49(10):2288–93. doi: 10.1161/STROKEAHA.118.021301 30355085

[39] Abulhasan YB, Al-Jehani H, Valiquette M-A, McManus A, Dolan-Cake M, Ayoub O, et al. Lumbar drainage for the treatment of severe bacterial meningitis. 2013;19(2):199–205. doi: 10.1007/s12028-013-9853-y 23739926

[40] Udy AA, Roberts JA, Lipman JJICM. Clinical implications of antibiotic pharmacokinetic principles in the critically ill. 2013;39(12):2070–82. doi: 10.1007/s00134-013-3088-4 24045886

[41] Morales-Alvarez MCJAICKD. Nephrotoxicity of antimicrobials and antibiotics. 2020;27(1):31–7. doi: 10.1053/j.ackd.2019.08.001 32146999

[42] Naughton CAJAFP. Drug-induced nephrotoxicity. 2008;78(6):743–50. 18819242

[43] Sales GTM, Foresto RDJRDAMB. Drug-induced nephrotoxicity. 2020;66:s82–s90. doi: 10.1590/1806-9282.66.S1.82 31939540

[44] Castelino R, Sathvik B, Parthasarathi G, Gurudev K, Shetty M, Narahari MJJocp, et al. Prevalence of medication‐related problems among patients with renal compromise in an Indian hospital. 2011;36(4):481–7. 10.1111/j.1365-2710.2011.01266.x 21535060

[45] Benninger F, Steiner IJJONT. Steroids in bacterial meningitis: yes. 2013;120(2):339–42. doi: 10.1007/s00702-012-0938-0 23238974

[46] van de Beek D, de Gans J, McIntyre P, Prasad KJTLID. Steroids in adults with acute bacterial meningitis: a systematic review. 2004;4(3):139–43. doi: 10.1016/S1473-3099(04)00937-5 14998499

[47] Abdinia B, Rezaee MA, Oskouie SAJIRCMJ. Etiology and antimicrobial resistance patterns of acute bacterial meningitis in children: a 10-year referral hospital-based study in northwest iran. 2014;16(7). doi: 10.5812/ircmj.17616 25237583 PMC4166102

[48] Hsia Y, Lee B, Versporten A, Yang Y, Bielicki J, Jackson C, et al. GARPEC and Global-PPS networks. Use of the WHO Access, Watch, and Reserve classification to define patterns of hospital antibiotic use (AwaRe): an analysis of paediatric survey data from 56 countries. 2019;7(7):e861–e71. 10.1016/S2214-109X(19)30071-3 31200888

[49] Adekoya I, Maraj D, Steiner L, Yaphe H, Moja L, Magrini N, et al. Comparison of antibiotics included in national essential medicines lists of 138 countries using the WHO Access, Watch, Reserve (AwaRe) classification: a cross-sectional study. 2021;21(10):1429–40. doi: 10.1016/S1473-3099(20)30854-9 34332706

[50] Rafi S, Rasheed H, Usman M, Nawaz HA, Anjum SM, Chaudhry M, et al. Availability of essential medicines in Pakistan—A comprehensive document analysis. 2021;16(7):e0253880. doi: 10.1371/journal.pone.0253880 34242249 PMC8270130

[51] Ata M, Hoque R, Biswas RSR, Mostafa A, Hasan FU, Barua HRJCM-O-SHMCJ. Antibiotics prescribing pattern at outpatient department of a tertiary medical college hospital. 2018;17(2):36–9.

[52] Nau R, Djukic M, Spreer A, Ribes S, Eiffert HJEROA-IT. Bacterial meningitis: an update of new treatment options. 2015. doi: 10.1586/14787210.2015.1077700 26293166

[53] Veltman JA, Bristow CC, Klausner JDJJOTIAS. Meningitis in HIV‐positive patients in sub‐Saharan Africa: a review. 2014;17(1):19184. doi: 10.7448/IAS.17.1.19184 25308903 PMC4195174

[54] Khan F, Rizvi M, Fatima N, Shukla I, Malik A, Khatoon RJNA. Bacterial meningitis in North India: Trends over a period of eight years. 2011;16(1).

[55] Chao Y-N, Chiu N-C, Huang F-YJJOM, Immunology, Infection. Clinical features and prognostic factors in childhood pneumococcal meningitis. 2008;41(1):48–53. 18327426

[56] Borhani-Haghighi A, Safari R, Heydari ST, Soleimani F, Sharifian M, Kashkuli SY, et al. Hospital mortality associated with stroke in southern Iran. 2013;38(4):314. 24293785 PMC3838983

[57] Wanleenuwat P, Suntharampillai N, Iwanowski PJS. Antibiotic-induced epileptic seizures: mechanisms of action and clinical considerations. 2020;81:167–74. doi: 10.1016/j.seizure.2020.08.012 32827980

[58] Pomar V, De Benito N, Mauri A, Coll P, Gurguí M, Domingo PJBid. Characteristics and outcome of spontaneous bacterial meningitis in patients with diabetes mellitus. 2020;20(1):1–9. doi: 10.1186/s12879-020-05023-5 32312231 PMC7171854

[59] Magazzini S, Nazerian P, Vanni S, Paladini B, Pepe G, Casanova B, et al. Clinical picture of meningitis in the adult patient and its relationship with age. 2012;7(4):359–64. doi: 10.1007/s11739-012-0765-1 22419148

[60] Chua TH, Xu Z, King NKKJBI. Neurological manifestations in COVID-19: a systematic review and meta-analysis. 2020;34(12):1549–68. doi: 10.1080/02699052.2020.1831606 33074036

[61] Fisher J, Linder A, Calevo MG, Bentzer PJCDOSR. Non‐corticosteroid adjuvant therapies for acute bacterial meningitis. 2021;(11). doi: 10.1002/14651858.CD013437.pub2 34813078 PMC8610076

[62] Proulx N, Frechette D, Toye B, Chan J, Kravcik SJQ. Delays in the administration of antibiotics are associated with mortality from adult acute bacterial meningitis. 2005;98(4):291–8. doi: 10.1093/qjmed/hci047 15760921

[63] Bekele F, Fekadu G, Bekele K, Dugassa D, Sori JJSOM. Drug-related problems among patients with infectious disease admitted to medical wards of Wollega University Referral Hospital: Prospective observational study. 2021;9:2050312121989625. doi: 10.1177/2050312121989625 33552517 PMC7841694

[64] Viktil KK, Blix HSJB, pharmacology c, toxicology. The impact of clinical pharmacists on drug‐related problems and clinical outcomes. 2008;102(3):275–80. 10.1111/j.1742-7843.2007.00206.x 18248511

