# Supplementary material for: Assessment of potential drug-related problems (PDRP) and clinical outcomes in bacterial meningitis patients admitted to tertiary care hospitals
Source: PLoS One. 2023 Oct 9;18(10):e0285171. doi: 10.1371/journal.pone.0285171 (PMC10561832; doi:10.1371/journal.pone.0285171)
Supplement: S1 File — (DOCX) [file pone.0285171.s002.docx]

**S1 File**

**Evidence based checklist for evaluation of bacterial meningitis patients**

**Patient demographics/ general information**

**GENDER/SEX:** 1. Male 2. Female

**AGE:**

1. Group A Peadiatrics

2. Group B Adults

3. Group C Geriatrics

**CLINICAL OUTCOME:** 1. Death/Expired 2. Discharged from Hospital

**HOSPITAL STAY (Days):** 1. 01-10 days 2. 11-20 days 3. 21-30 days

**HOSPITAL SECTOR:** 1. Public sector 2. Private sector

**DISEASE: Bacterial Meningitis**

**Organisms involved:** 1. Neisseria meningitidis 2. S. pneumonia 3. Streptococcus agalactiae

4. aerobic gram-negative bacilli 5. Listeria monocytogenes 6. Pseudumonas aeruginosa

7. E.coli 8. H Influenza

**NOSOCOMIAL INFECTIONS:** Yes No

**CULTURE SENSITIVITY TEST:** Yes No

**CSF** Yes No

**CT/MRI SCAN** YES NO

**DRUGS PRESCRIBED/ADMINISTERED:**

(Antibiotics, Antiviral, Antipyretic, Antifungal, Antiepileptics, statins and others) Others* PPIs, Antihypertensive, Antidiabetic etc.)_____________________________

____________________________________________________________________________

____________________________________________________________________________

**WARD OF ADMISSION:**

**VITAL SIGNS:**

1. Heart rate
2. Blood pressure
3. Respiratory rate
4. Temperature/Fever

**LABORATORY VALUES OF PATIENTS:**

**HB_______________________**

**RBC______________________**

**Platelets___________________**

**TWBC____________________**

**Electrolytes________________**

**Other lab values* PT/INR, CRP, UCE, LFT, LIPID PROFILE etc_____________________**

| Primary Domain | Cause | Frequency of DRPs |
| --- | --- | --- |
|  |  |  |
| Drug Selection | Inappropriate drug according to guidelines/formulary |  |
|  | Inappropriate combination of drugs, or drugs and herbal medications, or drugs and dietary supplements |  |
|  | Appropriate drug according to guidelines/formulary |  |
| Dose Selection | Dosage regimen not frequent enough |  |
|  | Dosage regimen too frequent |  |
|  | Dose timing instructions wrong, unclear or missing |  |
| Treatment Duration | Duration of treatment too short |  |
|  | Duration of treatment too long |  |
|  | Proper Duration of treatment |  |
| Dispensing | Prescribed drug not available |  |
|  | Necessary information not provided or incorrect advice provided |  |
| Drug use process | Inappropriate timing of administration or dosing intervals |  |
|  | Wrong drug administered |  |
|  | Appropriate timing of administration or dosing intervals |  |

***Above list is adapted from PCNE classification checklist. (Europe, 2017), (M. J. Farrukh, S. A. Hisham, & Z. A. J. A. J. P. S. Bin Zainal, 2014).**
